# Psychosocial and behavioral correlates of Mpox infection among men who have sex with men in China: a multicenter cross-sectional study with implications for health equity

**DOI:** 10.1186/s40249-026-01427-8

**Published:** 2026-02-18

**Authors:** Gang Xu, Changyuan Zhou, Yuxuan Hua, Shangbin Liu, Jianyu Chen, Ying Wang, Jiechen Zhang, Yong Cai

**Affiliations:** 1 Public Health Department, Hongqiao International Institute of Medicine, Tongren Hospital, Shanghai Jiao Tong University School of Medicine, Shanghai, 200025 China; 2 School of Public Health, Shanghai Jiao Tong University School of Medicine, Shanghai, 200025 China; 3 Institute of Child and Adolescent Health, School of Public Health, Peking University Health Science Center, Beijing, 100191 China; 4 College of Public Health, Shanghai University of Medicine and Health Sciences, Shanghai, 200025 China; 5 Dermatology Department, Tongren Hospital, Shanghai Jiao Tong University School of Medicine, Shanghai, 200025 China

**Keywords:** Mpox, MSM, Psychosocial correlates, Unprotected anal intercourse, Substance use, HIV, Stigma, China, Cross-sectional study, Health equity

## Abstract

**Background:**

Mpox (formerly known as monkeypox) has disproportionately affected men who have sex with men (MSM), yet evidence from East Asia on psychosocial and behavioral correlates is limited. We examined factors associated with Mpox infection among MSM in China, with attention to health equity considerations.

**Methods:**

A multi-center cross-sectional survey was conducted in six Chinese cities (November 2023–March 2024). Eligible MSM (≥ 18 years; male-to-male sexual contact in the past six months) completed an anonymous questionnaire capturing sociodemographics, Human Immunodeficiency Virus (HIV) infection status (along with other health statuses like hypertension, diabetes, and hyperlipidemia), unprotected anal intercourse (UAI), multiple sex partnerships (MSP), pre-sex substance use (alcohol and drugs), recent voluntary counseling and testing for HIV (VCT), and psychosocial measures (perceived discrimination and self-esteem). Mpox infection was ascertained by self-reported clinical diagnosis or syndromic criteria aligned with national guidance. Multivariable logistic regression estimated adjusted odds ratios (a*OR*) and 95% confidence intervals (*CI*). False discovery rate was controlled using the Benjamini–Hochberg procedure within prespecified hypothesis families.

**Results:**

Among 2403 participants (mean age 30.6 years), 5.9% met the Mpox case definition. In multivariable logistic regression analyses, younger men had the highest odds of Mpox infection. Using ages 18–24 years as the reference, older groups showed significantly lower odds: 25–34 years (*OR* = 0.508, 95% *CI* 0.317–0.814, *P* = 0.005), 35–44 years (*OR* = 0.142, 95% *CI* 0.064–0.316, *P* < 0.001), and ≥ 45 years (*OR* = 0.170, 95% *CI* 0.045–0.641, *P* = 0.009). Behavioral and health correlates that significantly increased the odds of Mpox included UAI (*OR* = 2.841, 95% *CI* 1.858–4.345, *P* < 0.001), MSP (*OR* = 1.586, 95% *CI* 1.063–2.368, *P* = 0.024), pre-sex drug use (*OR* = 2.359, 95% *CI* 1.199–4.639, *P* = 0.013), and pre-sex alcohol use (*OR* = 3.955, 95% *CI* 2.530–6.181, *P* < 0.001). Furthermore, recent VCT participation was positively associated with Mpox (*OR* = 2.079, 95% *CI* 1.19–3.632, *P* = 0.010). Psychosocial measures were significantly associated with Mpox infection, including higher perceived discrimination (*OR* = 1.136, 95% *CI* 1.026–1.258, *P* = 0.014) and lower self-esteem (*OR* = 0.962, 95% *CI* 0.929–0.997, *P* = 0.034).

**Conclusions:**

Mpox infection among MSM in China was associated with psychosocial (perceived discrimination, self-esteem) and behavioral (condomless anal sex, partner number, pre-sex substance use) characteristics, with higher odds observed among younger men. Integrating Mpox screening and risk-reduction support into existing HIV/sexually transmitted infection services, alongside anti-stigma and youth-focused outreach, may help address observed disparities.

**Supplementary Information:**

The online version contains supplementary material available at 10.1186/s40249-026-01427-8.

## Background

Since May 2022, Mpox (formerly known as monkeypox) has re-emerged as a global public health concern, with rapid spread beyond historical foci in West and Central Africa [1]. In July 2022, the World Health Organization (WHO) declared the outbreak a Public Health Emergency of International Concern (PHEIC) [2]. By August 2025, 162,785 laboratory-confirmed cases had been reported across 140 WHO member states, including 3531 cases in China [1]. Transmission has been predominantly sexual, with a high proportion of cases occurring among men who have sex with men (MSM) [1].

Although WHO lifted the initial PHEIC in May 2023, the emergence of a new clade (Clade Ib) associated with transmission in the Democratic Republic of the Congo prompted a renewed PHEIC declaration in August 2024 [3]. In the Chinese mainland, incident cases peaked in July 2023 and subsequently declined; however, the risk of sustained transmission persists in the context of large and heterogeneous MSM networks, estimated to include over eight million individuals nationwide [4, 5]. These epidemiological features underscore the need to identify correlates of infection to inform targeted prevention.

Beyond biological susceptibility, MSM face disproportionate structural and social burdens that can shape vulnerability to infection and access to care [6]. Stigma and discrimination—amplified in the context of sexually transmitted infections—have been documented among MSM and can adversely affect healthcare-seeking, mental health, and engagement with prevention services in China and elsewhere [7].

International evidence highlights heterogeneity in demographic correlates of Mpox infection; for example, age-related patterns have differed across Brazilian and Dutch cohorts [8, 9]. Occupational and socioeconomic factors have also been linked to sexual risk behaviors and sexually transmitted diseases (STDs) in other settings, warranting examination for Mpox in China [10]. In addition, MSM who are married to women may serve as potential “bridges” for transmission into heterosexual networks, raising further equity and family health concerns.

Psychosocial determinants are increasingly recognized as barriers to effective outbreak control. Stigma and perceived discrimination can reduce healthcare utilization, delay diagnosis, and increase the likelihood of undiagnosed infection [8, 11, 12]. These factors may also operate through psychological pathways—such as lowered self-esteem—associated with higher sexual risk-taking, thereby reinforcing transmission within marginalized networks [11, 12]. Despite their relevance, psychosocial correlates of Mpox infection remain understudied in Asian contexts, where cultural norms and service accessibility may differ from Western settings.

Behavioral correlates of Mpox infection have been consistently reported, including unprotected anal intercourse (UAI), multiple sexual partnerships (MSP), and pre-sex drug use [8, 9, 13, 14]. While emerging Chinese data have described certain risk behaviors among MSM with Mpox [15], comprehensive assessments of pre-sex drug and alcohol use, and the role of heterosexual contacts, remain limited. The intersection of Mpox with other STDs, particularly Human Immunodeficiency Virus (HIV) infection, also appears context dependent. Studies from Europe and the Americas suggest frequent co-occurrence of HIV, syphilis, and other STDs among Mpox cases [14–18], and recent HIV testing has been reported as independently associated with Mpox diagnosis in some cohorts, possibly reflecting risk-driven health-seeking [19]. However, limited Chinese studies indicate potentially distinct patterns, underscoring the need for larger, multi-site investigations [19].

To address these gaps, we conducted a multi-center cross-sectional study among MSM in six Chinese cities to examine psychosocial and behavioral correlates of Mpox infection. We focused on perceived discrimination and self-esteem as key psychosocial constructs, and on sexual practices and pre-sex substance use as behavioral domains.

## Methods

### Study design and participant recruitment

We conducted a nationwide, multi-center cross-sectional survey between November 2023 and March 2024 across six geographically diverse regions in the Chinese mainland selected using an established regional sampling framework [20]: Xinjiang (Northwest), Liaoning (Northeast), Shaanxi (Central), Yunnan (Southwest), Guangdong (Southeast), and Shanghai (East).

A multi-stage, non-probability sampling strategy was used. First, research sites were selected via convenience sampling in collaboration with local Centers for Disease Control and Prevention (CDCs) and Non-Governmental Organizations (NGOs) [21]. Given the concealed nature of the MSM population, participant recruitment at each site employed snowball sampling with multiple parallel chains. In each region, local NGOs engaged 5–10 MSM 'seed' participants based on eligibility and active social presence in diverse subgroups (by age, occupation, and social venues) to reduce homophily. Each participant could refer up to 3 peers from their personal networks using coded invitations. Referrals proceeded in parallel chains without a fixed number of waves; chains were allowed to terminate naturally if no further eligible participants were identified. Recruitment ceased when regional targets were reached or when two consecutive weeks yielded fewer than five new referrals (operational stopping rule). The final sample reflects the aggregation of all active chains across regions.

The minimum required sample size was determined using the formula:

$$N=\frac{{Z}^{2}\cdot P\cdot (1-P)}{{E}^{2}}$$

Sample size calculations were based on the expected prevalence of Mpox infection, which was estimated to be *P* = 0.73% according to existing research [22]. For a 95% confidence level (*CI*) (*Z* = 1.96) and a margin of error (*E*) of 0.5%, the initial sample size was calculated. To account for the increased variability associated with snowball sampling, a design effect (*DE*) of 2.0 was applied. Further accounting for an anticipated 10% non-response rate, the final required sample size was calculated to be 2476 participants [20]. Eventually, A total of 2481 questionnaires were ultimately collected, of which 2403 met quality control criteria and were included in the final analysis, yielding an effective response rate of 96.9%. The distribution of valid questionnaires was: Shanghai (*n* = 569), Guangdong (*n* = 500), Xinjiang (*n* = 320), Shaanxi (*n* = 199), Yunnan (*n* = 313), and Liaoning (*n* = 502) [20].

Eligibility criteria for participation included: (1) assigned male at birth; (2) aged 18 years or older; (3) continuous residence in one of the six selected sites for a minimum of 6 months prior to enrollment; (4) reported male-to-male sexual contact within the past 6 months [18]. We excluded participants who completed the questionnaire in less than 300 s, made errors in quality control questions, and had IP addresses that were inconsistent with the survey site. This study guaranteed anonymity, and participants had the right to withdraw at any time without any consequences. Prior to conducting the study, all participants obtained written informed consent [17].

### Data collection and quality control

The survey instrument was developed through a rigorous process involving iterative consultations with public health experts and incorporation of validated scales. The excellent reliability and validity of this scale have been rigorously evaluated [23]. Prior to implementation, the questionnaire underwent pilot testing with MSM community members to optimize its cultural appropriateness, comprehensibility, and acceptability. All participants provided informed consent before study enrollment.

Data collection was conducted at NGO-operated sites that ensured participant privacy. Trained research assistants guided participants through the survey process, which utilized a web-based questionnaire platform (Wenjuanxing: https://www.wjx.cn/) accessible via QR code scanning. This anonymous data collection method was designed to minimize social desirability bias while maintaining data quality. The questionnaire required approximately 30 min to complete. To maximize response rates and compensate for time invested, participants received 80 CNY (approximately 11 USD) upon completion. The study protocol and procedures were approved by the Ethics Committee of Shanghai University of Medicine & Health Sciences (2023-MSMMpox-22-310222197604080237). Clinical trial number: not applicable.

Post-collection data management involved comprehensive cleaning procedures, including identification and treatment of outliers, assessment of missing data patterns, and verification of logical consistency, with appropriate statistical adjustments applied where necessary.

### Variables and measures

#### Demographic characteristics

Demographic characteristics included age, educational attainment (junior highs school and below, senior high school, college and above), marital status (single, married, divorced/widowed), occupational category (student, employed, unemployed/retired), monthly income (CNY ≤ 3000, 3001–6000, 6001–12,000, ≥ 12,001), and length of residence (local, < 5 years, ≥ 5 years).

#### Health status

Health status assessments included self-reported HIV infection status and STD status. The presence of specific chronic diseases was also assessed, specifically hypertension, diabetes, and hyperlipidemia.

#### Psychosocial factors

The study measured multiple psychosocial factors. Discrimination experiences were assessed using a 5-item scale [24] measuring both nonphysical and physical discrimination related to sexual identity. Binary responses (yes/no) were summed, with higher scores indicating greater exposure to discrimination (range: 0–5, Cronbach's *α* = 0.876). Self-esteem was measured using the validated Chinese version of the Rosenberg Self-Esteem Scale (RSES) [25, 26]. This 10-item instrument employs a 4-point Likert scale (1 = strongly disagree to 4 = strongly agree), with higher total scores indicating higher levels of self-esteem (range: 10–40, Cronbach's *α* = 0.872).

#### Behavioral factors

Behavioral measures encompassed sexual risk behaviors and HIV-related factors. Sexual risk behaviors in the preceding six months included MSP, UAI, sexual contact with women, unprotected vaginal intercourse (UVI), pre-sex drug use, and pre-sex alcohol use. HIV-related factors comprised self-reported HIV infection status and recent participation in voluntary counseling and testing for HIV (VCT).

#### Outcome measure

The primary outcome was Mpox infection, determined through both self-reported clinical diagnosis and syndromic assessment. The syndromic assessment included characteristic rash (genital or extremities), rash morphological features, associated flu-like symptoms, lymphadenopathy, and known exposure to diagnosed Mpox cases among sexual partners. Case definition criteria [27] classified participants as Mpox-positive if they either had a confirmed diagnosis from a healthcare institution or met the clinical criteria for suspected Mpox based on symptom presentation.

### Statistical analysis

All analyses were conducted in SPSS version 27.0 (IBM Corp., Armonk, NY, USA). Descriptive statistics were generated for all study variables. Continuous variables were summarized as mean and standard deviation (*SD*), and categorical variables as frequencies and percentages. Bivariate associations with Mpox infection were assessed using the Wilcoxon rank-sum test for continuous variables and Pearson’s *χ*^*2*^ test (or Fisher’s exact test when expected cell counts were < 5) for categorical variables. To identify independent correlates of Mpox infection, we fitted hierarchical multivariable logistic regression models. To conceptualize potential causal pathways and guide the selection of adjustment sets for multivariable analysis, a Conceptual Directed Acyclic Graph (DAG) was constructed (Supplementary Figure S1). Based on the resulting DAG, the minimal sufficient adjustment set for analysis included age, education level, marital status, monthly income, and occupation. Additionally, hypertension, diabetes, and hyperlipidemia were included to account for potential differences in healthcare utilization. Three sequential models were specified. Model 1 included demographic characteristics (age, educational level, marital status, monthly income, and occupation) and health status indicators. Model 2 additionally incorporated behavioral factors (VCT, MSP, UAI, sex with women, pre-sex substance use). Model 3 further included all variables from Model 2 along with psychosocial factors (discrimination and self-esteem). Results are presented as odds ratios (*OR*) with 95% *CI*. To address multiplicity, we controlled the false discovery rate (FDR) using the Benjamini–Hochberg (BH) procedure within prespecified families of hypotheses (psychosocial, behavioral, and subgroup comparisons). Unless otherwise specified, all reported *P*-values in the manuscript are two-sided and unadjusted; corresponding BH FDR-adjusted *Q*-values are provided in Supplementary Material (Tables S4–S6). Statistical significance was defined as *P* < 0.05. Key associations remained consistent at a threshold of *Q* < 0.05.

## Results

### Characteristics of participants

The final analytical sample comprised 2403 participants. The mean age was 30.59 years (*SD* = 8.02), with the majority (51.1%, *n* = 1228) aged 25–34 years, followed by those aged 18–24 years (23.5%, *n* = 564), 35–44 years (19.1%, *n* = 459), and ≥ 45 years (6.3%, *n* = 152). The sample was highly educated, with 78.6% (*n* = 1888) having completed college education or above, while 14.9% (*n* = 358) had completed senior high school, and 6.5% (*n* = 157) had junior high school education or below. Additional sociodemographic characteristics of the study population are detailed in Table 1. (See Table 1 in additional files).

**Table 1:** Sociodemographic and clinical characteristics of the study participants by Mpox infection status

	Total (*N* %)	Mpox (*n* %)		*P* value
	(*N* = 2403)	No infection (*n* = 2261)	Infected (*n* = 142)	
Age, years	30.59 ± 8.02	30.81 ± 8.07	27.10 ± 6.16	< 0.001
Age group				
18–24	564 (23.5)	507 (22.4)	57 (40.1)	< 0.001
25–34	1228 (51.1)	1157 (51.2)	71 (50.0)	
35–44	459 (19.1)	448 (19.8)	11 (7.7)	
≥ 45	152 (6.3)	149 (6.6)	3 (2.0)	
Education level				0.750
≤ Junior high school	157 (6.5)	146 (6.5)	11 (7.7)	
Senior high school	358 (14.9)	339 (15.0)	19 (13.4)	
≥ College	1888 (78.6)	1776 (78.5)	112 (78.9)	
Marital status				0.018
Single	2035 (84.7)	1920 (84.9)	115 (81.0)	
Married	255 (10.6)	231 (10.2)	24 (16.9)	
Divorced/widowed	113 (4.7)	110 (4.9)	3 (2.1)	
Employment				0.483
Mental labor	386 (16.1)	365 (16.1)	21 (14.8)	
Manual labor	774 (32.2)	723 (32.0)	51 (35.9)	
Student	320 (13.3)	304 (13.4)	16 (11.3)	
Freelance work	813 (33.8)	762 (33.7)	51 (35.9)	
Unemployed	110 (4.6)	107 (4.7)	3 (2.1)	
Length of residence				0.358
Local	936 (39.0)	874 (38.7)	62 (43.7)	
< 5 year	870 (36.2)	819 (36.2)	51 (35.9)	
≥ 5 years	597 (24.8)	568 (25.1)	29 (20.4)	
Sexual orientation				0.114
Homosexual	1887 (78.5)	1783 (78.9)	104 (73.2)	
Others	516 (21.5)	478 (21.1)	38 (26.8)	
HIV				< 0.001
No	2204 (91.7)	2091 (92.5)	113 (79.6)	
Yes	199 (8.3)	170 (7.5)	29 (20.4)	
Hypertension				< 0.001
No	2248 (93.5)	2125 (94.0)	123 (86.6)	
Yes	155 (6.5)	136 (6.0)	19 (13.4)	
Diabetes				0.002
No	2334 (97.1)	2202 (97.4)	132 (93.0)	
Yes	69 (2.9)	59 (2.6)	10 (7.0)	
Hyperlipidemia				0.040
No	2181 (90.8)	2059 (91.1)	122 (85.9)	
Yes	222 (9.2)	202 (8.9)	20 (14.1)	
Metabolic status				0.003
Healthy	2078 (86.5)	1967 (87.0)	111 (78.2)	
Unhealthy	325 (13.5)	294 (13.0)	31 (21.8)	

### Distribution of mpox infections by population characteristics

Of the 2403 participants, 142 (5.9%) reported Mpox infection, among which 56 (39.4%) had laboratory-confirmed diagnoses and 86 (60.6%) were diagnosed through syndrome assessment. HIV infection was reported by 199 (8.3%) individuals. Sexual risk behaviors were common in the sample: nearly half (49.8%, *n* = 1197) reported multiple sexual partnerships in the preceding six months, 41.9% engaged in UAI, and among the 16.9% who reported vaginal sex, 59.7% did not use protection. Pre-sex drug use was reported by 2.9% of participants, while pre-sex alcohol use was observed in a significantly larger proportion (37.2%). A history of sexually transmitted infections was reported by 9.2% (*n* = 221) of participants.

Bivariate analyses revealed significant demographic, behavioral and psychosocial differences (Table 1) between participants with and without Mpox infection. Mpox infection rates varied significantly across age groups (*P* < 0.001) and marital status (*P* = 0.018), with higher prevalence among married participants (16.9% vs. 10.2%). HIV co-infection was more frequent among those with Mpox (20.4%) compared to those without (7.5%).

Participants with Mpox infection reported significantly higher rates of sexual risk behaviors compared to those without: MSP (64.1% vs. 48.9%, *P* = 0.001), UAI (74.6% vs. 39.9%, *P* < 0.001), sexual contact with women (38.7% vs. 15.6%, *P* < 0.001), and UVI (80.0% vs. 56.5%, *P* = 0.002). Pre-sex drug use (16.9% vs. 2.0%, *P* < 0.001) and alcohol use (78.2% vs. 34.6%, *P* < 0.001) before sexual activity were also more prevalent among those with Mpox infection.

Psychological measures showed significant differences between groups. Wilcoxon rank-sum tests indicated that individuals with Mpox infection had significantly lower self-esteem scores and higher perceived discrimination levels compared to those without infection (both *P* < 0.001).

### Associations between Mpox infection and psychosocial and behavioral factors

#### Model 1: Multivariable model with social and disease covariates

Model 1 included demographic characteristics and basic health conditions (Table 2). Age showed a statistical association with Mpox infection. Compared to the 18–24 years reference group, the 25–34 years (*OR* = 0.368, 95% *CI* 0.239–0.565), 35–44 years (*OR* = 0.089, 95% *CI* 0.042–0.187), and ≥ 45 years (*OR* = 0.047, 95% *CI* 0.013–0.174) groups were associated with lower odds of infection. Marital status was also associated with Mpox infection (reference group: single), with being married (*OR* = 3.400, 95% *CI* 1.950–5.928) related to higher odds of infection. HIV infection was associated with Mpox infection (*OR* = 2.853, 95% *CI* 1.767–4.606). Occupation (manual work: *OR* = 1.356, 95% *CI* 0.774–2.374; student: *OR* = 0.620, 95% *CI* 0.265–1.448; freelance work: *OR* = 1.220, 95% *CI* 0.697–2.134; unemployed: *OR* = 0.538, 95% *CI* 0.145–1.994), education level (senior high school: *OR* = 0.679, 95% *CI* 0.294–1.566; college or above: *OR* = 0.744, 95% *CI* 0.351–1.578), income (3001–6000 CNY: *OR* = 1.215, 95% *CI* 0.648–2.277; 6001–12,000 CNY: *OR* = 1.459, 95% *CI* 0.748–2.844; ≥ 12,001 CNY: *OR* = 1.577, 95% *CI* 0.683–3.639), hypertension (*OR* = 1.605, 95% *CI* 0.802–3.211), diabetes (*OR* = 2.038, 95% *CI* 0.818–5.077), and hyperlipidemia (*OR* = 1.464, 95% *CI* 0.770–2.785) were not statistically significant in this model.

**Table 2:** Multivariable logistic regression analysis of associations between sociodemographic factors, health conditions, and Mpox infection [Model 1, *N* = 2403, *OR* (95% *CI*)]

Variables	*OR*	95% *CI*		*P*-Value
		Lower 95% *CI*	Upper 95% *CI*	
Age				
25–34 years	0.368	0.239	0.565	< 0.001
35–44 years	0.089	0.042	0.187	< 0.001
45– years	0.047	0.013	0.174	< 0.001
Occupation				
Manual work	1.356	0.774	2.374	0.287
Student	0.620	0.265	1.448	0.269
Freelance work	1.220	0.697	2.134	0.487
Unemployed	0.538	0.145	1.994	0.353
Marital status				
Married	3.400	1.950	5.928	< 0.001
Divorced or widowed	1.006	0.297	3.414	0.992
Education level				
Senior high school	0.679	0.294	1.566	0.364
≥ College	0.744	0.351	1.578	0.441
Income				
3001–6000 CNY	1.215	0.648	2.277	0.544
6001–12,000 CNY	1.459	0.748	2.844	0.267
≥ 12,001 CNY	1.577	0.683	3.639	0.286
HIV infection status	2.853	1.767	4.606	< 0.001
Hypertension	1.605	0.802	3.211	0.181
Diabetes	2.038	0.818	5.077	0.126
Hyperlipidemia	1.464	0.770	2.785	0.245

Reference categories were: age 18–24 years; mental labor; single; education level ≤ junior school; monthly income ≤ 3000 CNY; HIV negative; no hypertension; no diabetes; no hyperlipidemia; *P*-values are two-sided and uncorrected; the corresponding *Q*-values after Benjamini–Hochberg (BH) procedure adjustment are presented in Supplementary Tables S4 named Benjamini-Hochberg (BH) Adjusted *P*-values (*Q*-values) in Model 1. *OR* odd ratio, *CI* confidence interval, *CNY* Chinese Yuan

#### Model 2: Multivariable model additionally adjusted for behavioral covariates

Model 2 added sexual behavior and health service-related variables to Model 1. The association between age and infection remained significant, with *OR*s of 0.470 (25–34 years), 0.129 (35–44 years), and 0.141 (≥ 45 years). Occupation and marital status showed overall statistical associations. Specifically, compared with men engaged in mental labor, being a student was associated with lower odds (*OR* = 0.388, 95% *CI* 0.158–0.951). Meanwhile, compared with single men, being married was associated with higher odds (*OR* = 2.092, 95% *CI* 1.097–3.989). Regarding behavioral factors, receiving voluntary counseling and testing for HIV (VCT) in the past 6 months (*OR* = 2.034, 95% *CI* 1.170–3.535), having multiple sex partnerships (MSP) (*OR* = 1.637, 95% *CI* 1.100–2.436), engaging in unprotected anal intercourse (UAI) (*OR* = 2.915, 95% *CI* 1.914–4.440), having sexual contact with women (*OR* = 1.631, 95% *CI* 1.019–2.612), pre-sex alcohol use (*OR* = 4.080, 95% *CI* 2.620–6.356), and pre-sex drug use (*OR* = 2.491, 95% *CI* 1.268–4.891) were all associated with higher odds of infection. The association for HIV infection was attenuated (*OR* = 1.698, 95% *CI* 0.986–2.925). Education level (senior high school: *OR* = 0.696, 95% *CI* 0.288–1.680; ≥ college: *OR* = 0.792, 95% *CI* 0.363–1.726), monthly income (3001–6000 CNY: *OR* = 0.728, 95% *CI* 0.377–1.405; 6001–12,000 CNY: *OR* = 0.750, 95% *CI* 0.374–1.506; ≥ 12,001 CNY: *OR* = 0.819, 95% *CI* 0.344–1.949), and the underlying chronic conditions hypertension (*OR* = 1.138, 95% *CI* 0.519–2.496), diabetes (*OR* = 1.297, 95% *CI* 0.448–3.755), and hyperlipidemia (*OR* = 1.471, 95% *CI* 0.741–2.920)were not statistically significant (Table 3).

**Table 3:** Multivariable logistic regression analysis of associations between social-behavioral factors and Mpox infection [Model 2, *N* = 2403, *OR* (95% *CI*)]

Variables	*OR*	95% *CI*		*P*-Value
		Lower 95% *CI*	Upper 95% *CI*	
Age				
25–34 years	0.470	0.295	0.747	0.001
35–44 years	0.129	0.059	0.285	< 0.001
45– years	0.141	0.038	0.524	0.003
Occupation				
Manual work	1.462	0.799	2.675	0.218
Student	0.388	0.158	0.951	0.039
Freelance work	1.252	0.685	2.291	0.465
Unemployed	0.593	0.143	2.467	0.473
Marital status				
Married	2.092	1.097	3.989	0.025
Divorced or widowed	0.520	0.139	1.940	0.330
Education level				
Senior high school	0.696	0.288	1.680	0.420
≥ College	0.792	0.363	1.726	0.557
Income				
3001–6000 CNY	0.728	0.377	1.405	0.345
6001–12,000 CNY	0.750	0.374	1.506	0.419
≥ 12,001 CNY	0.819	0.344	1.949	0.652
HIV infection status	1.698	0.986	2.925	0.056
Hypertension	1.138	0.519	2.496	0.747
Diabetes	1.297	0.448	3.755	0.631
Hyperlipidemia	1.471	0.741	2.920	0.270
VCT	2.034	1.170	3.535	0.012
MSP	1.637	1.100	2.436	0.015
UAI	2.915	1.914	4.440	< 0.001
Sex with women	1.631	1.019	2.612	0.042
Pre-sex alcohol use	4.080	2.620	6.356	< 0.001
Pre-sex drug use	2.491	1.268	4.891	0.008

Reference categories were: age 18–24 years; mental labor; single; education level ≤ junior school; monthly income ≤ 3000 CNY; HIV negative; no hypertension; no diabetes; no hyperlipidemia; no voluntary counseling and testing for HIV (VCT) in the past 6 months; no multiple sex partnerships (MSP) in the past 6 months; no unprotected anal intercourse (UAI) in the past 6 months; no sexual contact with women in the past 6 months; no pre-sex alcohol use in the past 6 months; no pre-sex drug use in the past 6 months. *P*-values are two-sided and uncorrected; the corresponding *Q*-values after Benjamini–Hochberg (BH) procedure adjustment are presented in Supplementary Tables S5 named Benjamini-Hochberg (BH) Adjusted *P*-values (*Q*-values) in Model 2. *OR* odd ratio, *CI* confidence interval, *CNY *Chinese Yuan

#### Model 3: Multivariable model additionally adjusted for psychological covariates

Model 3 further incorporated psychosocial variables into Model 2 (Table 4). The association with age remained significant, with *OR*s of 0.508 (25–34 years), 0.142 (35–44 years), and 0.170 (≥ 45 years). Among behavioral factors, VCT (*OR* = 2.079, 95% *CI* 1.190–3.632), MSP (*OR* = 1.586, 95% *CI* 1.063–2.368), UAI (*OR* = 2.841, 95% *CI* 1.858–4.345), pre-sex alcohol use (*OR* = 3.955, 95% *CI* 2.530–6.181), and pre-sex drug use (*OR* = 2.359, 95% *CI* 1.199–4.639) were associated with higher odds, with pre-sex alcohol use showing the strongest association. For psychosocial factors, each one-unit increase in the self-esteem score was associated with slightly lower odds of infection (*OR* = 0.962, 95% *CI* 0.929–0.997). Each one-unit increase in the discrimination score was associated with higher odds (*OR* = 1.136, 95% *CI* 1.026–1.258). The associations for HIV status and sexual contact with women were of borderline significance (*P* = 0.061 and *P* = 0.063, respectively) (Table 4). The main conclusions remained consistent after FDR control. Occupation (manual work: *OR* = 1.369, 95% *CI* 0.744–2.519; student: *OR* = 0.436, 95% *CI* 0.177–1.073; freelance work: *OR* = 1.228, 95% *CI* 0.669–2.257; unemployed: *OR* = 0.548, 95% *CI* 0.130–2.317), marital status (married: *OR* = 1.923, 95% *CI* 0.999–3.700; divorced/widowed: *OR* = 0.525, 95% *CI* 0.141–1.953), education level (senior high school: *OR* = 0.785, 95% *CI* 0.321–1.918; ≥ college: *OR* = 0.875, 95% *CI* 0.397–1.929), monthly income (3001–6000 CNY: *OR* = 0.812, 95% *CI* 0.420–1.571; 6001–12,000 CNY: *OR* = 0.856, 95% *CI* 0.424–1.727; ≥ 12,001 CNY: *OR* = 0.987, 95% *CI* 0.409–2.378), and the underlying chronic conditions hypertension (*OR* = 1.051, 95% *CI* 0.477–2.313), diabetes (*OR* = 1.474, 95% *CI* 0.514–4.229), and hyperlipidemia (*OR* = 1.321, 95% *CI* 0.654–2.665)were not statistically significant in this final model. In the sensitivity analysis using only laboratory-confirmed cases as the outcome, the direction and magnitude of the associations were largely consistent; however, due to the reduced sample size, some *OR*s had wider confidence intervals. The results of the sensitivity analysis are provided in the Supplementary Materials (Tables S1. Sensitivity analysis of sociodemographic-disease factors and Mpox infection [Model 1, *N* = 2403, *OR* (95% *CI*)]. Table S2. Sensitivity analysis of social-behavioral factors and Mpox infection [Model 2, *N* = 2403, *OR* (95% *CI*)]. Table S3. Sensitivity analysis of psycho-social-behavioral factors and Mpox infection [Model 3, *N* = 2403, *OR* (95% *CI*)]).

**Table 4:** Multivariable logistic regression analysis of associations between psycho-social-behavioral factors and Mpox infection [Model 3, *N* = 2403, *OR* (95% *CI*)]

Variables	*OR*	95% *CI*		*P*-Value
		Lower 95% *CI*	Upper 95% *CI*	
Age				
25–34 years	0.508	0.317	0.814	0.005
35–44 years	0.142	0.064	0.316	< 0.001
45– years	0.170	0.045	0.641	0.009
Occupation				
Manual work	1.369	0.744	2.519	0.312
Student	0.436	0.177	1.073	0.071
Freelance work	1.228	0.669	2.257	0.507
Unemployed	0.548	0.130	2.317	0.414
Marital status				
Married	1.923	0.999	3.700	0.050
Divorced or widowed	0.525	0.141	1.953	0.336
Education level				
Senior high school	0.785	0.321	1.918	0.595
≥ College	0.875	0.397	1.929	0.741
Income				
3001–6000 CNY	0.812	0.420	1.571	0.536
6001–12,000 CNY	0.856	0.424	1.727	0.664
≥ 12,001 CNY	0.987	0.409	2.378	0.976
HIV infection status	1.688	0.977	2.916	0.061
Hypertension	1.051	0.477	2.313	0.902
Diabetes	1.474	0.514	4.229	0.470
Hyperlipidemia	1.321	0.654	2.665	0.438
VCT	2.079	1.190	3.632	0.010
MSP	1.586	1.063	2.368	0.024
UAI	2.841	1.858	4.345	< 0.001
Sex with women	1.566	0.977	2.511	0.063
Pre-sex alcohol use	3.955	2.530	6.181	< 0.001
Pre-sex drug use	2.359	1.199	4.639	0.013
Self-esteem score	0.962	0.929	0.997	0.034
Discrimination score	1.136	1.026	1.258	0.014

Reference categories were: age 18–24 years; mental labor; single; education level ≤ junior school; monthly income ≤ 3000 CNY; HIV negative; no hypertension; no diabetes; no hyperlipidemia; no voluntary counseling and testing for HIV (VCT) in the past 6 months; no multiple sex partnerships (MSP) in the past 6 months; no unprotected anal intercourse (UAI) in the past 6 months; no sexual contact with women in the past 6 months; no pre-sex alcohol use in the past 6 months; no pre-sex drug use in the past 6 months. Discrimination and self-esteem scores were modeled as continuous variables, the *OR* represents the change in the odds of Mpox infection for every 1-unit increase in the score. *P*-values are two-sided and uncorrected; the corresponding *Q*-values after Benjamini–Hochberg (BH) procedure adjustment are presented in Supplementary Tables S6 named Benjamini-Hochberg (BH) Adjusted *P*-values (*Q*-values) in Model 3. *OR* odd ratio, *CI* confidence interval, *CNY* Chinese Yuan

## Discussion

In this multi-center cross-sectional study of 2403 MSM across six regions in China, 5.9% met the case definition for Mpox. This proportion exceeds the 0.73% reported by national surveillance in August 2023 [22], likely reflecting our broader case definition that included syndromically compatible, suspected cases and may have captured infections among individuals who did not access care. Given that sexual contact is the predominant transmission route in China [27], delineating psychosocial and behavioral correlates is essential for tailoring prevention in this key population.

Sociodemographic correlates showed a consistent age gradient: younger MSM (18–24 years) had higher odds of Mpox relative to older groups, whereas men aged ≥ 45 years had lower odds. This observed pattern in older adults is potentially attributable to residual protection from historical smallpox vaccination [28, 29]. However, our finding that 18–24 year-old face the highest odds contrasts with global surveillance data from the WHO, which often places the highest burden outside Africa within the 30–39 age group [1]. This highlights a critical and specific vulnerability in the Chinese MSM youth demographic. This pattern may reflect age-related HIV transmission dynamics among young MSM [30, 31]. While students showed lower odds of infection in the intermediate model (Model 2), this association was not significant in the fully adjusted model or sensitivity analyses. This instability suggests that occupational status is likely a proxy for other underlying determinants rather than an independent correlate of Mpox infection. The observed fluctuation might stem from selection bias (e.g., higher accessibility of students to NGO-led recruitment) or unmeasured heterogeneity within the student population, further emphasizing the need to focus on specific behavioral and psychosocial drivers over broad demographic categories. Regarding marital status, married MSM exhibited higher odds of Mpox in models 1 and 2, consistent with prior research [22]. This suggests complex sexual networks involving both male and female partners and possible bridge dynamics with implications for household and partner health. Although this association was attenuated after adjusting for psychosocial factors in Model 3, suggesting potential mediation by variables like self-esteem, it remained significant in the sensitivity analysis restricted to confirmed cases. This highlights the robustness of the finding and the necessity for future research with precise outcomes to clarify these complex relationships.

The observed intersection with HIV warrants attention. Approximately one in five participants with Mpox reported living with HIV, lower than global estimates (38–50%) [32] but suggestive of overlapping risk networks. Notably, while HIV infection was significantly associated with Mpox in the sociodemographic model (Model 1), this association was attenuated and lost statistical significance after adjusting for sexual behaviors (Models 2 and 3). This pattern suggests that the elevated risk initially observed among people living with HIV might be partly explained by the co-occurrence of high-risk sexual behaviors within this subgroup, rather than HIV status acting as an independent predictor in the final model. However, the cross-sectional design precludes definitive distinction between mediation and confounding pathways. Future longitudinal or event-level investigations are therefore required to rigorously disentangle these specific mechanisms. Nevertheless, existing literature indicates that immunosuppression resulting from HIV infection can significantly exacerbate the clinical severity and adverse outcomes of Mpox [32, 33]. This underscores the importance of integrating Mpox prevention and management into HIV care, as well as sustainable focus on this vulnerable population in future preventive strategies against Mpox.

Behavioral correlates were aligned with existing literature. MSP and UAI were associated with Mpox [22, 28], and, among married MSM, recent heterosexual contact emerged as an additional correlate, echoing concerns about bridging into heterosexual networks [34]. pre-sex substance use, especially alcohol, showed relatively stronger associations, consistent with literature linking substance-involved sex to impaired judgment, reduced condom use, and event-level risk escalation [35, 36]. While these findings support targeted risk-reduction around pre-sex substance use, interpretation should consider potential residual confounding by unmeasured event or venue characteristics (e.g., group sex, sauna/club attendance, polysubstance use) [36]. The absence of dose–response data on substance use limits inferences regarding intensity thresholds for risk. The positive association between recent voluntary counseling and testing (VCT) and Mpox likely reflects reverse causation (where symptoms or perceived risk prompt testing) or confounding by risk-seeking behaviors [37]. Additionally, VCT participation reflects greater engagement with health services, which increases the probability of case ascertainment and contributes to detection bias. Programmatically, this suggests that Mpox screening and counseling could be feasibly embedded within HIV/STD services to leverage existing points of contact.

Psychosocial correlates, specifically higher perceived discrimination and lower self-esteem, were independently associated with Mpox. These constructs plausibly operate through multiple pathways: delayed care-seeking and diagnosis due to stigma [38, 39], reduced engagement with prevention services, and elevated sexual risk behaviors linked to psychological distress [40]. Given consistent evidence linking stigma to poorer infectious disease control outcomes, these findings reinforce the importance of integrating anti-stigma and mental health components into Mpox prevention for MSM in China.

Our findings underscore clear health disparities in the Chinese MSM population. To operationalize health equity, interventions should address the specific vulnerabilities identified in our study. This requires two core strategic actions: First, services should be tailored for high-risk cohorts such as younger MSM (18–24 years) and married individuals (who may face heightened stigma and lower service access). Second, it is essential to integrate psychological support and anti-stigma efforts to mitigate the structural effects of discrimination and low self-esteem. Furthermore, this robust framework should prioritize resource allocation to these structurally vulnerable groups, offering confidential, accessible, and context-specific outreach [41].

Guided by this health equity framework, our findings support multi-level, equity-oriented prevention strategies that prioritize youth-focused risk reduction by delivering sex-positive, age-appropriate education and accessible Mpox information for MSM aged 18–24 years, including event-level strategies that address substance-involved sex (such as pre-planning condom availability, setting limits on alcohol use, buddy systems, and post-event testing reminders). In addition, services for married MSM should be confidential and nonjudgmental, explicitly addressing bisexual concurrency and offering partner services—including voluntary partner notification, testing, and protection for female partners where appropriate. Mpox prevention and care should be integrated into existing HIV/STD platforms by co-locating symptom screening, brief risk counseling, and referral within VCT and STD clinics, while leveraging joint messaging and, where feasible, combined screening panels to capitalize on established service touchpoints. Finally, psychosocial support and anti-stigma efforts are essential to minimize care avoidance. Brief screening for discrimination-related distress and low self-esteem should be embedded within sexual health services with warm referral pathways to counseling and peer support. To ensure safe access, services must guarantee confidential or anonymous testing options and provide discreet partner services tailored for married MSM. These clinical efforts should be complemented by community-level anti-stigma campaigns co-developed with community-based organizations (CBOs) or NGOs to foster non-stigmatizing communication and improve timely presentation for testing and treatment. These strategies are aligned with the WHO's "leave no one behind" principle and national HIV/STD program guidance. By providing context-specific evidence from an East Asian setting, our study sheds light on integrated Mpox prevention and control strategies. Future implementation studies are warranted to evaluate their feasibility, acceptability, and impact specifically in the identified priority subgroups.

Several limitations should be considered. First, the cross-sectional design precludes causal inference; temporality is uncertain for associations between behaviors, psychosocial constructs, and Mpox. The VCT–Mpox association may reflect reverse causation (risk- or symptom-driven testing), and infection itself could heighten perceived discrimination or reduce self-esteem. Second, recruitment via CDC/NGO networks and snowball sampling likely favored individuals engaged with services or those within large, homogeneous social networks. This may have resulted in the overrepresentation of specific subgroups while potentially underrepresenting harder-to-reach MSM, thereby limiting generalizability. Third, behavioral data were self-reported and subject to recall and social desirability biases; although syndromic criteria broadened case capture, laboratory confirmation was not universal. Fourth, despite the geographic diversity of our study sites, the resulting sample sizes were non-proportionally distributed across the six regions, reflecting variations in local recruitment capacity. Moreover, as our multivariable models did not incorporate region fixed effects, potential residual spatial confounding cannot be ruled out. Therefore, extrapolation to other regions or cultural contexts should be cautious. Fifth, exposure measures were coarse: a six-month recall window and binary coding of pre-sex substance use without quantity, frequency, or event timing constrain dose–response and temporality assessments and leave residual confounding by event-level and network factors. Furthermore, research designs should shift to event-level designs with quantity–frequency assessment, capture venue and group-sex participation, and integrate biological confirmation. Additionally, prospective studies are needed to clarify temporality and causal pathways among the observed correlates.

## Conclusions

In this multi-center cross-sectional study of MSM in China, Mpox infection was associated with psychosocial characteristics (higher perceived discrimination, lower self-esteem) and behavioral factors (UAI, MSP, pre-sex substance use), with higher odds observed among younger men and those living with HIV. These patterns indicate potential for ongoing transmission within specific subgroups and service touchpoints, underscoring the value of equity-oriented, targeted approaches. Integrated programs that combine sex-positive education, accessible and timely testing, linkage to care, and embedded psychosocial support—implemented within existing HIV/STD platforms and complemented by anti-stigma initiatives—may help address observed disparities in access and outcomes. Given the cross-sectional design, the reported associations should not be interpreted causally; prospective studies with biological confirmation and event-level behavioral measurement are warranted to clarify temporality and transmission pathways and to evaluate the effectiveness of integrated prevention strategies.

## Supplementary Information

Supplementary Material 1.

## Data Availability

The datasets used and/or analyzed during the current study are available from the corresponding author on reasonable request.

## Abbreviations

AIDS: Acquired immunodeficiency syndrome
BFI: Big Five Inventory
BH: Benjamini–Hochberg
CBO: Community-based organization
CDC: Centers for Disease Control and Prevention
*CI*: Confidence interval
DAG: Directed Acyclic Graph
DE: Design effect
FDR: False discovery rate
HIV: Human Immunodeficiency Virus
MSM: Men who have sex with men
MSP: Multiple sex partnership
NGO: Non-governmental organization
*OR*: Odds ratio
PHEIC: Public Health Emergency of International Concern
RSES: Rosenberg Self-Esteem Scale
*SD*: Standard deviation
STD: Sexually transmitted disease
UAI: Unprotected anal intercourse
UVI: Unprotected vaginal intercourse
VCT: Voluntary Counseling and Testing, for HIV
WHO: World Health Organization
