# Supplementary material for: Psychosocial and behavioral correlates of Mpox infection among men who have sex with men in China: a multicenter cross-sectional study with implications for health equity
Source: Infect Dis Poverty. 2026 Feb 18;15:25. doi: 10.1186/s40249-026-01427-8 (PMC12914930; doi:10.1186/s40249-026-01427-8)
Supplement: Supplementary file 1 — Supplementary Material 1. [file 40249_2026_1427_MOESM1_ESM.docx]

**Supplementary material**


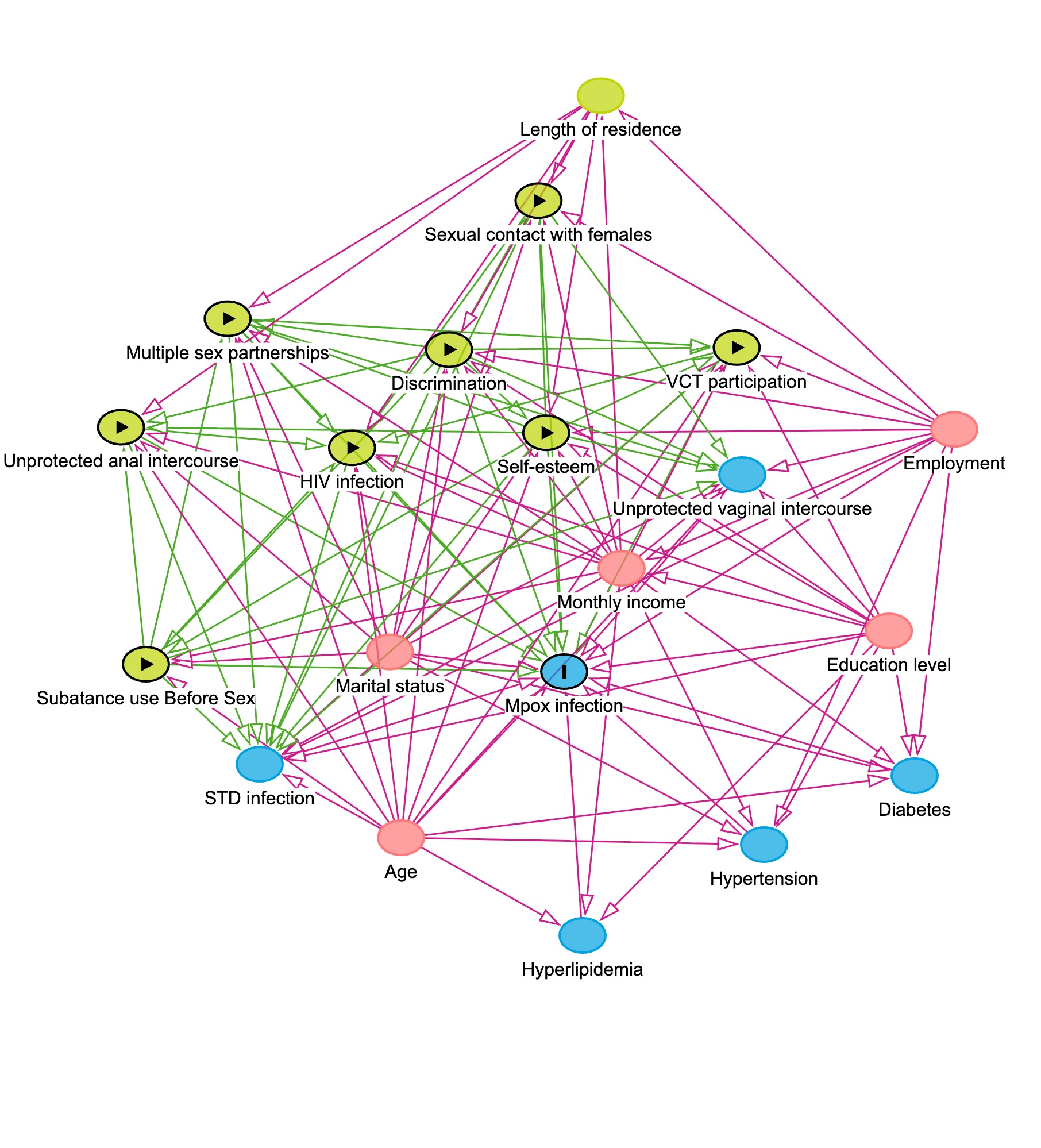


**Fig S1. Conceptual Directed Acyclic Graph (DAG) illustrating the assumed causal structure for Mpox infection in MSM**

Note: This DAG represents the hypothesized causal structure and adjustment strategy for multivariable analysis. VCT: Voluntary Counseling and Testing for HIV; STD: sexually transmitted disease.

**Table S1. Sensitivity analysis of sociodemographic-disease factors and Mpox infection [Model 1, *N* = 2403, *OR* (95% *CI*)]**

| Variables | *OR* | 95% CI | | *P*-Value |
| --- | --- | --- | --- | --- |
|  |  | Lower 95% *CI* | Upper 95% *CI* |  |
| **Age** |  |  |  | ＜0.001 |
| 25–34 years | 0.320 | 0.164 | 0.624 | 0.001 |
| 35–44 years | 0.041 | 0.011 | 0.155 | ＜0.001 |
| 45– years | 0.048 | 0.009 | 0.245 | ＜0.001 |
| **Occupation** |  |  |  | 0.694 |
| Manual work | 1.063 | 0.440 | 2.573 | 0.891 |
| Student | 0.931 | 0.241 | 3.591 | 0.917 |
| Freelance work | 1.340 | 0.566 | 3.170 | 0.506 |
| Unemployed | 2.426 | 0.543 | 10.845 | 0.246 |
| **Marital status** |  |  |  | ＜0.001 |
| Married | 9.221 | 4.369 | 19.463 | ＜0.001 |
| Divorced or widowed | 1.268 | 0.158 | 10.158 | 0.823 |
| **Education level** |  |  |  | 0.596 |
| Senior high school | 0.510 | 0.136 | 1.910 | 0.318 |
| ≥ College | 0.716 | 0.232 | 2.208 | 0.560 |
| **Income** |  |  |  | 0.084 |
| 3001–6000 CNY | 2.340 | 0.784 | 6.981 | 0.128 |
| 6001–12,000 CNY | 4.049 | 1.300 | 12.607 | 0.016 |
| ≥ 12,001 CNY | 4.081 | 1.021 | 16.308 | 0.047 |
| **HIV infection status** | 4.205 | 2.146 | 8.242 | ＜0.001 |
| **Hypertension** | 1.229 | 0.415 | 3.638 | 0.710 |
| **Diabetes** | 1.422 | 0.358 | 5.653 | 0.617 |
| **Hyperlipidemia** | 1.807 | 0.698 | 4.675 | 0.223 |

Notes: Reference categories were: age 18–24 years; mental labor; single; education level ≤ junior school; monthly income ≤ 3000 CNY; no hypertension; no diabetes; no hyperlipidemia;

**Table S2. Sensitivity analysis of social-behavioral factors and Mpox infection [Model 2, *N* = 2403, *OR* (95% CI)]**

| Variables | *OR* | 95% CI | | *P*-Value |
| --- | --- | --- | --- | --- |
|  |  | Lower 95% *CI* | Upper 95% *CI* |  |
| **Age** |  |  |  | 0.004 |
| 25–34 years | 0.425 | 0.201 | 0.898 | 0.025 |
| 35–44 years | 0.076 | 0.018 | 0.321 | ＜0.001 |
| 45– years | 0.272 | 0.047 | 1.579 | 0.147 |
| **Occupation** |  |  |  | 0.075 |
| Manual work | 1.566 | 0.572 | 4.285 | 0.383 |
| Student | 0.428 | 0.095 | 1.925 | 0.269 |
| Freelance work | 1.837 | 0.679 | 4.972 | 0.231 |
| Unemployed | 5.056 | 0.838 | 30.502 | 0.077 |
| **Marital status** |  |  |  | 0.006 |
| Married | 4.022 | 1.620 | 9.985 | 0.003 |
| Divorced or widowed | 0.427 | 0.040 | 4.589 | 0.482 |
| **Education level** |  |  |  | 0.479 |
| Senior high school | 0.411 | 0.094 | 1.787 | 0.236 |
| ≥ College | 0.687 | 0.214 | 2.209 | 0.529 |
| **Income** |  |  |  | 0.557 |
| 3001–6000 CNY | 1.200 | 0.357 | 4.039 | 0.768 |
| 6001–12,000 CNY | 1.774 | 0.511 | 6.162 | 0.367 |
| ≥ 12,001 CNY | 2.251 | 0.505 | 10.030 | 0.287 |
| **HIV infection status** | 1.996 | 0.910 | 4.374 | 0.084 |
| **Hypertension** | 0.525 | 0.140 | 1.963 | 0.338 |
| **Diabetes** | 0.885 | 0.189 | 4.150 | 0.877 |
| **Hyperlipidemia** | 2.013 | 0.735 | 5.514 | 0.173 |
| **VCT** | 3.248 | 1.164 | 9.065 | 0.024 |
| **MSP** | 0.864 | 0.453 | 1.647 | 0.657 |
| **UAI** | 4.236 | 1.929 | 9.302 | ＜0.001 |
| **Sex with women** | 1.915 | 0.924 | 3.970 | 0.081 |
| **Drink alcohol before sex** | 5.688 | 2.325 | 13.913 | ＜0.001 |
| **Use drugs before sex** | 6.793 | 2.833 | 16.287 | ＜0.001 |

Notes: Reference categories were: age 18–24 years; mental labor; single; education level ≤ junior school; monthly income ≤ 3000 CNY; no voluntary counseling and testing for HIV (VCT) in the past 6 months; HIV negative; no multiple sex partnerships (MSP) in the past 6 months; no unprotected anal intercourse (UAI) in the past 6 months; no sexual contact with women in the past 6 months; no pre-sex alcohol use in the past 6 months; no pre-sex drug use in the past 6 months; no hypertension; no diabetes; no hyperlipidemia.

**Table S3. Sensitivity analysis of psycho-social-behavioral factors and Mpox infection [Model 3, *N* = 2403, *OR* (95% CI)]**

| Variables | *OR* | 95% CI | | *P*-Value |
| --- | --- | --- | --- | --- |
|  |  | Lower 95% *CI* | Upper 95% *CI* |  |
| **Age** |  |  |  | 0.007 |
| 25–34 years | 0.462 | 0.217 | 0.982 | 0.045 |
| 35–44 years | 0.080 | 0.019 | 0.346 | 0.001 |
| 45– years | 0.375 | 0.064 | 2.176 | 0.274 |
| **Occupation** |  |  |  | 0.128 |
| Manual work | 1.466 | 0.530 | 4.055 | 0.461 |
| Student | 0.493 | 0.108 | 2.243 | 0.360 |
| Freelance work | 1.814 | 0.664 | 4.960 | 0.246 |
| Unemployed | 4.979 | 0.825 | 30.041 | 0.080 |
| **Marital status** |  |  |  | 0.018 |
| Married | 3.486 | 1.385 | 8.774 | 0.008 |
| Divorced or widowed | 0.486 | 0.047 | 5.031 | 0.545 |
| **Education level** |  |  |  | 0.520 |
| Senior high school | 0.453 | 0.102 | 2.012 | 0.298 |
| ≥ College | 0.801 | 0.246 | 2.606 | 0.713 |
| **Income** |  |  |  | 0.434 |
| 3001–6000 CNY | 1.403 | 0.422 | 4.663 | 0.581 |
| 6001–12,000 CNY | 2.131 | 0.617 | 7.356 | 0.231 |
| ≥ 12,001 CNY | 2.772 | 0.623 | 12.340 | 0.181 |
| **HIV infection status** | 1.973 | 0.893 | 4.360 | 0.093 |
| **Hypertension** | 0.485 | 0.130 | 1.810 | 0.282 |
| **Diabetes** | 1.061 | 0.224 | 5.024 | 0.941 |
| **Hyperlipidemia** | 1.816 | 0.643 | 5.131 | 0.260 |
| **VCT** | 3.374 | 1.194 | 9.530 | 0.022 |
| **MSP** | 0.792 | 0.412 | 1.523 | 0.485 |
| **UAI** | 4.168 | 1.875 | 9.265 | ＜0.001 |
| **Sex with women** | 1.861 | 0.899 | 3.853 | 0.094 |
| **Drink alcohol before sex** | 5.316 | 2.159 | 13.089 | ＜0.001 |
| **Use drugs before sex** | 6.559 | 2.695 | 15.964 | ＜0.001 |
| **Self-esteem score** | 0.961 | 0.905 | 1.021 | 0.201 |
| **Discrimination score** | 1.231 | 1.030 | 1.472 | 0.022 |

Notes: Reference categories were: age 18–24 years; mental labor; single; education level ≤ junior school; monthly income ≤ 3000 CNY; no voluntary counseling and testing for HIV (VCT) in the past 6 months; HIV negative; no multiple sex partnerships (MSP) in the past 6 months; no unprotected anal intercourse (UAI) in the past 6 months; no sexual contact with women in the past 6 months; no pre-sex alcohol use in the past 6 months; no pre-sex drug use in the past 6 months; no hypertension; no diabetes; no hyperlipidemia. Discrimination and self-esteem scores were modeled as continuous variables, the *OR* represents the change in the odds of Mpox infection for every 1-unit increase in the score.

**Table S4. Benjamini-Hochberg (BH) Adjusted *P*-values (*Q*-values) in Model 1**

| Variables | *Q*-Value |
| --- | --- |
| **Age** |  |
| 25–34 years | <0.001 |
| 35–44 years | <0.001 |
| 45– years | <0.001 |
| **Occupation** |  |
| Manual work | 0.431 |
| Student | 0.431 |
| Freelance work | 0.548 |
| Unemployed | 0.467 |
| **Marital status** |  |
| Married | <0.001 |
| Divorced or widowed | 0.992 |
| **Education level** |  |
| Senior high school | 0.467 |
| ≥ College | 0.529 |
| **Income** |  |
| 3001–6000 CNY | 0.576 |
| 6001–12,000 CNY | 0.431 |
| ≥ 12,001 CNY | 0.431 |
| **HIV infection status** | <0.001 |
| **Hypertension** | 0.431 |
| **Diabetes** | 0.379 |
| **Hyperlipidemia** | 0.431 |
|  |  |

**Table S5. Benjamini-Hochberg (BH) Adjusted *P*-values (*Q*-values) in Model 2**

| Variables | *Q*-Value |
| --- | --- |
| **Age** |  |
| 25–34 years | 0.008 |
| 35–44 years | <0.001 |
| 45– years | 0.017 |
| **Occupation** |  |
| Manual work | 0.402 |
| Student | 0.091 |
| Freelance work | 0.567 |
| Unemployed | 0.567 |
| **Marital status** |  |
| Married | 0.067 |
| Divorced or widowed | 0.517 |
| **Education level** |  |
| Senior high school | 0.560 |
| ≥ College | 0.637 |
| **Income** |  |
| 3001–6000 CNY | 0.517 |
| 6001–12,000 CNY | 0.560 |
| ≥ 12,001 CNY | 0.680 |
| **HIV infection status** | 0.112 |
| **Hypertension** | 0.747 |
| **Diabetes** | 0.680 |
| **Hyperlipidemia** | 0.464 |
| **VCT** | 0.040 |
| **MSP** | 0.046 |
| **UAI** | <0.001 |
| **Sex with women** | 0.091 |
| **Drink alcohol before sex** | <0.001 |
| **Use drugs before sex** | 0.032 |

**Table S6. Benjamini-Hochberg (BH) Adjusted *P*-values (*Q*-values) in Model 3**

| Variables | *Q*-Value |
| --- | --- |
| **Age** |  |
| 25–34 years | 0.032 |
| 35–44 years | <0.001 |
| 45– years | 0.044 |
| **Occupation** |  |
| Manual work | 0.541 |
| Student | 0.131 |
| Freelance work | 0.659 |
| Unemployed | 0.632 |
| **Marital status** |  |
| Married | 0.119 |
| Divorced or widowed | 0.547 |
| **Education level** |  |
| Senior high school | 0.703 |
| ≥ College | 0.803 |
| **Income** |  |
| 3001–6000 CNY | 0.664 |
| 6001–12,000 CNY | 0.750 |
| ≥ 12,001 CNY | 0.976 |
| **HIV infection status** | 0.125 |
| **Hypertension** | 0.938 |
| **Diabetes** | 0.644 |
| **Hyperlipidemia** | 0.632 |
| **VCT** | 0.044 |
| **MSP** | 0.069 |
| **UAI** | <0.001 |
| **Sex with women** | 0.125 |
| **Drink alcohol before sex** | <0.001 |
| **Use drugs before sex** | 0.045 |
| **Self-esteem score** | 0.088 |
| **Discrimination score** | 0.045 |
